# Reconfigurable Light Imaging in Photonic Higher-Order Topological Insulators

**DOI:** 10.3390/nano12050819

**Published:** 2022-02-28

**Authors:** Xiaomeng Zhang, Yuyu Zhou, Xiaochen Sun, Xiujuan Zhang, Ming-Hui Lu, Yan-Feng Chen

**Affiliations:** 1 National Laboratory of Solid State Microstructures, Department of Materials Science and Engineering, Nanjing University, Nanjing 210093, China; mf20340083@smail.nju.edu.cn (X.Z.); mf20340087@smail.nju.edu.cn (Y.Z.); xcsun@nju.edu.cn (X.S.); yfchen@nju.edu.cn (Y.-F.C.); 2 Jiangsu Key Laboratory of Artificial Functional Materials, Nanjing 210093, China; 3 Collaborative Innovation Center of Advanced Microstructures, Nanjing University, Nanjing 210093, China

**Keywords:** higher-order topological insulators, anti-chiral edge states

## Abstract

Topological phases of matter with robust edge states have revolutionized the fundamental intuitions for wave control. The recent development of higher-order topological insulators (HOTIs) realizes even lower dimensional topological states that enable versatile wave manipulations (e.g., light imaging). However, in conventional HOTIs, the topological states are usually protected by certain crystalline symmetries and therefore bounded at specific locations, hindering their applications in modern digital ears, which often prefer tunability and reconfigurability. Here, we report the reconfigurable light imaging based on topological corner states and anti-chiral edge states in a two-dimensional (2D) photonic HOTI with a honeycomb lattice of yttrium iron garnet (YIG, a ferrite material) rods. Sublattices A and B are applied with magnetic fields in opposite directions, which realize the so-called modified Haldane model that hosts anti-chiral edge modes. By further breaking the lattice’s inversion symmetry via adjusting the radii of A and B rods, topological edge states with valley degrees of freedom emerge, which not only exhibit valley-dependence but also surprisingly show anti-chiral behaviors. In the valley edge gap, which is of nontrivial higher-order topology, corner states appear. With different combinations of corner states and anti-chiral edge states, versatile reconfigurable light imaging can be realized. As examples, a multiplexing waveguide-resonator device, a pine tree imaging that can be lit up or put out at will and selective imaging for partial objects in a two-heart pattern are demonstrated. The proposed HOTI shows high potential in future intelligent devices with exciting tunable and reconfigurable functions, which may inspire a wide range of applications such as topological switching, imaging processing, and nonreciprocal integrated photonics.

## 1. Introduction

In recent decades, topology in physics has drawn great attention and stimulated large research enthusiasm in both the scientific and technological communities [1,2,3,4,5] due to the topologically robust edge states for backscattering-immune channeling of electrons. In particular, chiral edge modes represent a type of edge state in systems with broken time reversal symmetry [6,7,8]. In strip geometry, the edge modes counter-propagate on the upper and lower edges, showing the chiral behavior. This type of edge state has a wide range of advantages for its particular robustness against non-magnetic disorders and defects [9,10,11,12]. Recently, a new type of edge state, known as the anti-chiral edge state, has been put forward, which can propagate in the same direction on opposite edges [13]. This interesting phenomenon was originally proposed in a so-called modified Haldane model and soon spread across many physical systems, for example, an exciton-polariton honeycomb lattice with strip geometry [14], a Heisenberg ferromagnet on the honeycomb lattice [15], a gyromagnetic photonic crystal (PC) [16,17], circuit lattices [18], and acoustics systems [19]. It has broad potential applications in integrated photonic devices such as non-reciprocal transmission.

Normally, *d*-dimensional (*d*D) topological insulators have *d*D gapped bulk states and (*d* − 1)D gapless boundary states. The recently discovered HOTIs, on the other hand, host even lower-dimensional gapless boundary states, opening another chapter for topological phases of matters, which have also attracted tremendous attention across the fields of condensed matter physics, optics, acoustics, etc. [20,21,22,23,24,25,26,27,28,29,30,31]. Using the HOTIs and their lower-dimensional boundary states, various wave control methods are proposed, for example, multi-dimensional topological switching [5], valley-selective corner sates [32,33,34,35], topological sub-wavelength imaging [36,37,38], and even controllable emergence of corner states enabled by non-linearity [39], voltage [40], or temperature [41]. Despite these celebrated advances, the HOTI-enabled functionalities often highly depend on the lattice geometries due to the fact that nontrivial higher-order topology is usually protected by the crystalline symmetries, which vary from lattice to lattice. As a result, the above discussed functionalities lack the flexibility of tunability and reconfigurability.

In this work, we report the reconfigurable light imaging based on topological corner states, incorporating anti-chiral edge states, in a 2D photonic crystal (PC) of the modified Haldane model with broken inversion symmetry. In the modified Haldane model, the two triangular sublattices are magnetically biased in opposite directions, tilting the Dirac points at the high symmetric points K and K′. The resultant edge states on the top and bottom edges acquire the same group velocity and become anti-chiral. By further breaking the inversion symmetry, valley-dependent edge states emerge, whose gap carries nontrivial higher-order band topology, and therefore gives rise to topological corner states. By incorporating corner states with external anti-chiral edge ports, we demonstrate versatile light imaging that can be tuned and reconfigured by flipping the magnetic fields. The remainder of the paper is organized as follows. The materials and methods are elaborated in Section 2. The photonic HOTI, its associated anti-chiral edge states, and the emergence of topological corner states are introduced in Section 3. In Section 4, we study various light imaging based on the 0D corner states and the 1D edge states. Finally, the conclusions are given in Section 5.

## 2. Materials and Methods

### 2.1. Materials

In this work, we considered the transverse-electric waves (i.e., the electric field is out of plane) and adopted a gyromagnetic material of the relative permeability tensor $\tilde{\mu }=\left[\begin{array}{ccc} \mu_{r} & i\mu_{k} & 0\\ -i\mu_{k} & \mu_{r} & 0\\ 0 & 0 & 1 \end{array}\right]$ with $\mu_{r}=1+\frac{\left(\omega_{0}+i\alpha \omega \right)\omega_{m}}{{\left(\omega_{0}+i\alpha \omega \right)}^{2}-\omega^{2}}$, $\mu_{k}=\frac{\omega \omega_{m}}{{\left(\omega_{0}+i\alpha \omega \right)}^{2}-\omega^{2}}$. Here, $\omega_{m}=\gamma M_{s}$ and $\omega_{0}=\gamma H$ with $\gamma =1.76\times 10^{11}s^{-1}T^{-1}$ as the gyromagnetic ratio, $\;M_{s}=1750$ Oe as the magnetization intensity, and *H* as the external magnetic field. *ω* denotes the operating frequency. *α* is the damping coefficient. In the main text, without loss of generality, we neglected the dissipation of the YIG (i.e., *α* = 0) and considered the dispersionless $\mu_{r}$ and $\mu_{k}$ at 14.5 GHz, under the static magnetic field $H_{\pm }=\pm 2200\;$ G. The dispersive $\mu_{r}$ and $\mu_{k}$ can be obtained following the above equations for each *ω*. 

### 2.2. Numerical Simulations

**Figure 1:**
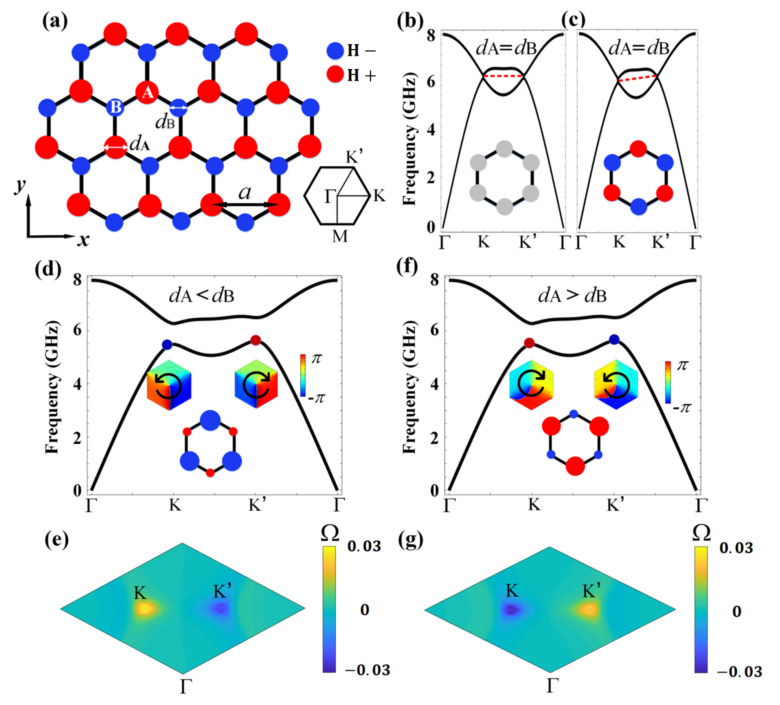
The proposed 2D PC with biased magnetic fields and broken inversion symmetry. (**a**) Schematic of the PC, which is a honeycomb lattice of sublattices A and B, with $d_{A}$ ($d_{B}$ ) the radius of A (B) rods. A positive magnetic field $H_{+}$ was applied on A rods, while a negative magnetic field H_  was applied on B rods. The inset shows the first Brillouin zone. (**b**) Band structure for the PC with $d_{A}=d_{B}$, where no magnetic field was applied. The red dashed line connects the two Dirac points at the high symmetric points K and K′. (**c**) The same as (**b**), only with biased magnetic fields. (**d**) Band structure with $d_{A}<d_{B}$ (which is denoted as PC1). The insets show the phase distributions of the electric fields for the lowest band at K and K′, with the arrows indicating the phase vortices. (**e**) Berry curvature distributions of the lowest band for the PC in (**d**). (**f**–**g**) The same as (**d**–**e**), only for the PC with $d_{A}>d_{B}$ (which is denoted as PC2).

**Figure 2:**
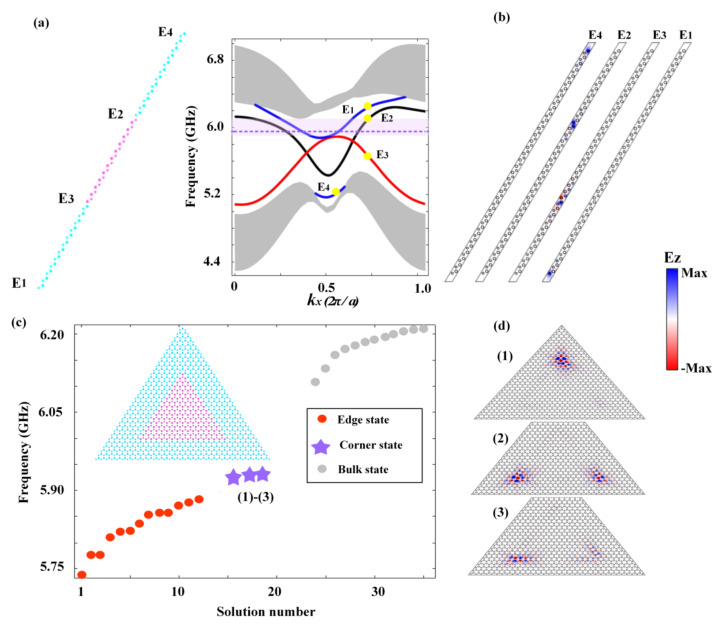
Anti-chiral edge states and the associated topological corner states. (**a**) Left panel: A strip supercell composed of PC1 (blue regions) and PC2 (pink region), which presents four edges marked by E_1_, E_2_, E_3_, and E_4_. Right panel: Projected band structures of the strip supercell. The colored curves represent the four edge states corresponding to E_1_–E_4_ while the grey shadings denote bulk states. The edge gap for E_3_ edge states is highlighted by the light red shading, which carries nontrivial high-order topology and therefore supports topological corner states, as schematically indicated by the purple dash line. (**b**) The electric field distributions of the four anti-chiral edge states marked in (**a**). (**c**) The eigenspectra of a triangular box supercell composed of PC2 surrounded by PC1 (forming E_3_-type edges and geometric corners, as shown by the inset). Grey, red, and purple symbols indicate the bulk states, the E_3_ valley edge states, and the in-gap corner states, respectively. (**d**) Electric field distributions of the three in-gap corner states labeled in (**c**).

**Figure 3:**
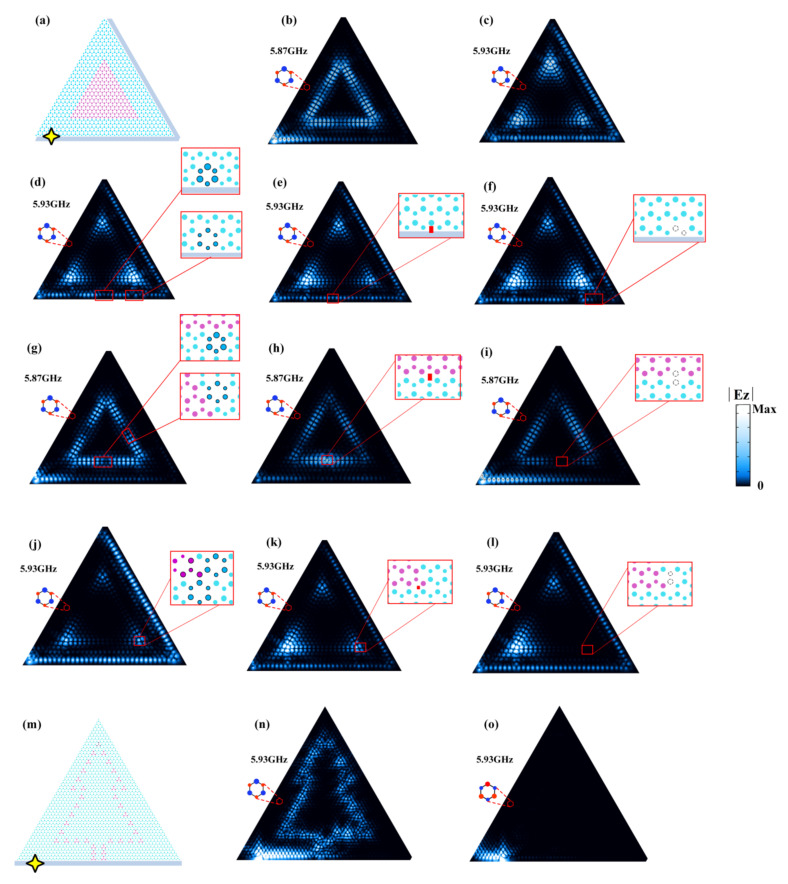
A multiplexing waveguide-resonator device and a tunable pine tree imaging with E_4_-edges as control ports. (**a**) Schematic of a triangular whisper-gallery ring composed of PC2 enclosed by PC1 and attached to an E_4_ edge port. The excitation is employed at the left port-entrance, as indicated by the yellow star. Numerically simulated electric field distributions of the waveguide-resonator in (**a**) at the exciting frequencies of (**b**) 5.87 GHz and (**c**) 5.93 GHz are presented. The insets depict the magnetic bias (with red color indicating the positive magnetic field and the blue color indicating the negative magnetic field). (**d**–**l**) Robustness study for the three types of topological states including (**d**–**f**) the anti-chiral edge states induced by breaking the time-reversal symmetry, (**g**–**i**) the valley-dependent edge states induced by breaking the inversion symmetry and (**j**–**l**) the corner states induced by breaking the inversion symmetry. Three types of perturbations are deliberately introduced, which are, successively from left to right, the $C_{3v}$-preserving expanded/shrunken unit cells (highlighted by darker colors and solid circles), inserted dielectric obstructions (indicated by the red blocks), and the $C_{3v}$ -breaking removed PC rods (whose locations are indicated by the dotted circles). The expanding/shrinking strength in (**d**,**g**) is 10% while that in (**j**) is 5%. The dielectric constant for the obstructions is 12 while their geometric sizes are 0.24a×0.6a for (**e**) and (**h**) and 0.18a×0.4a for (**k**). (**m**) Schematic of a complex image of a pine tree constructed by the PC2 pixels as coding units and PC1 as the background. An E_4_ edge port is also attached to the bottom of the image. (**n**) Electric field distributions of the pine tree image lit up by the incoming signal from the left port-entrance. (**o**) The same as (**n**), only that the magnetic fields are flipped.

**Figure 4:**
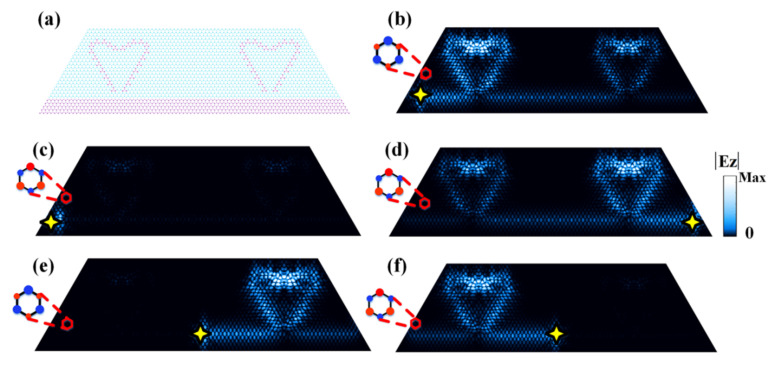
Versatile reconfigurable light imaging in a two-heart complex pattern with E_2_-edges as control ports. (**a**) Schematic of the two heart coding shapes that are connected to each other by an E_2_-edge port. (**b**) Electric field distributions for the two-heart light imaging illuminated by an incoming signal from the left port-entrance (denoted by the yellow star). (**c**) The same as (**b**), only that the magnetic fields are flipped. (**d**) The same as (**c**), only the excitation is launched from the right port-entrance. (**e**–**f**) The partial heart imaging enabled by excitations at the center of the edge port, with either the right heart lit up or the left heart lit up upon different magnetic biases. The operating frequency is 5.93 GHz for all simulations in (**b**–**f**).

The band structures, edge/corner dispersions, and the excitation color maps throughout our work were obtained by conducting numerical simulations using the commercial finite element method solver (COMSOL Multiphysics). In generating the band structures in Figure 1b–d,f, a unit cell was adopted with the boundaries imposed by periodic boundary conditions. In generating the edge dispersions in Figure 2a, a ribbon structure was adopted, consisting of a slice of PC2 sandwiched by two slices of PC1. The upper and lower boundaries were bounded by air channels (with trivial topology) while the left and right were imposed as periodic boundary conditions. To obtain the data in Figure 2c, we considered a triangular finite structure composed of PC2 enclosed by PC1. The outer boundaries were set as radiating boundary conditions. In the simulations in Figure 3, the triangular structures and the complex imaging patterns were constructed by PC1 (blue regions) and PC2 (pink regions) with careful cell positioning. The excitation ports were composed of PC1 and air channels. In Figure 3b–l, the outermost lower and right boundaries were set as hard boundary conditions (i.e., as the perfect electric conductors) while the outermost left boundary was set as the radiating boundary condition. In Figure 3n,o, the outermost lower boundary was set as the hard boundary condition while the rest were set as the radiating boundary conditions. In the simulations in Figure 4, all the outermost boundaries were set as radiating boundary conditions.

## 3. Topological Properties of the Proposed HOTI 

We considered a honeycomb lattice of YIG rods as shown in Figure 1a, consisting of two triangular sublattices A and B (colored in red and blue, respectively). $d_{A}$ and $d_{B}$ denote the radii of the YIG rods for A and B, respectively. a is the lattice constant. The permittivity of YIG can be taken as ε=15 while its permeability is described by a tensor [7]

(1) $$\mu =\left(\begin{array}{ccc} \mu_{r} & i\mu_{k} & 0\\ -i\mu_{k} & \mu_{r} & 0\\ 0 & 0 & 1 \end{array}\right).$$

Depending on different magnetic fields, the diagonal and off-diagonal terms $\mu_{r}$ and $\mu_{k}$ can take different values [42]. For zero magnetic field, YIG behaves as a regular dielectric material with $\mu_{r}=1$ and $\mu_{k}=0$. Here, without loss of generality, we fixed $H_{\pm }=\pm 2200\;$G for the positive and negative magnetic fields, which give $\mu_{r}=0.84$ and $\mu_{k}=\pm 0.41$ [17]. 

The honeycomb lattice obeys the $C_{6v}$ symmetry and hosts Dirac points at the high symmetric points K and K′ in the momentum space (see the band structure in Figure 1b, where the TE-polarized waves are considered). Here, $d_{A}=d_{B}$ is taken as 0.15a with a=1.5 cm. Without magnetic bias (i.e., H=0), the Dirac points at K and K′ are located at the same frequency. Upon open boundaries, the system exhibits dispersionless edge states connecting the inequivalent Dirac points (as schematically illustrated by the dashed line in Figure 1b), in a similar way as the Fermi-arc in semi-metals [43,44,45]. When magnetic fields are applied on sublattice A and B with opposite directions (with $H_{+}$ applied to A and $H_{-}$ applied to B), the strong anisotropy tilts the Dirac points, with the one at the K point moving down and the other at the K′ point moving up (see Figure 2c). The resultant edge states both acquire a positive group velocity as schematically illustrated by the dashed line in Figure 2c, leading to the anti-chiral behavior. 

When different radii of A and B are considered, the inversion symmetry of the honeycomb lattice is broken, with $C_{6v}$ symmetry reduced to $C_{3v}$ symmetry. As a consequence, the Dirac degeneracies at K and K′ points are lifted and a band gap is opened, as exemplified by the band structure in Figure 1d with $d_{A}=0.28a$ and $d_{B}=0.36a$. We present the phase distributions for the electric field at K and K′ points in the insets of Figure 1d, which show opposite vortices (i.e., counterclockwise at the K point and clockwise at the K′ point), suggesting the wave functions at these two symmetric points carry opposite angular momenta. Such an interesting phenomenon gives rise to the well-known quantum valley Hall effect [46,47]. To characterize the nontrivial band topology, we further calculated the Berry curvature distribution in the k space (i.e., $\Omega_{n}\left(k\right)=\nabla_{k}\times A_{n}\left(k\right))$, where $A_{n}\left(k\right)=i\langle u_{n}\left(k\right)\left|\frac{\partial }{\partial k}\right|u_{n}\left(k\right)\rangle$ is the Berry connection, with $u_{n}\left(k\right)$ representing the periodic parts of the wave functions for the nth band below the band gap (in our case, it was the lowest band). The results are shown in Figure 1e, which exhibited opposite distributions at K and K′ points, consistent with the vortex distributions. The integration of the Berry curvature over the entire Brillion zone represents the Chern number. The opposite distributions of Ω at the K and K′ points indicate vanishing Chern number. When only considering the integration over the K (K′) neighbor, however, Ω led to the non-zero value of $\frac{1}{2}$ ($-\frac{1}{2}$), which is commonly referred to as the valley-polarized Chern number and is used to characterize the nontrivial valley Hall effect. 

The valley Hall effect enables valley-dependent edge states. To investigate the valley edge states, a physical interface needs to be constructed where the lattice symmetry is preserved so that no inter-valley scattering is generated. In order to do so, we considered another PC with $d_{A}=0.36a$ and $d_{B}=0.28a$. To distinguish this PC from that in Figure 1d, we refer to the former as PC2 and the latter as PC1. The band structure for PC2 is presented in Figure 1f, which shows the same dispersions as that in Figure 1d (i.e., the band structure for PC1). However, very different behaviors were observed for the phase distributions at the K and K′ points, with the former showing a clockwise vortex and the latter showing a counterclockwise vortex. This was in contrast to the vortices for PC1, indicating a clear band inversion associated with the topological phase transition. Similar Berry curvature distributions were calculated for PC2, as shown in Figure 1g. Again, Ω showed opposite distributions at the K and K′ points. However, unlike that for PC1, Ω at K′ (K) became positive (negative), indicating a non-zero valley-polarized Chern number of $\frac{1}{2}$ ($-\frac{1}{2}$). This suggests that PC1 and PC2 are indeed in different topological phases and can therefore form a physical interface to investigate the valley edge states.

Next, we considered a strip geometry consisting of PC1 and PC2, as illustrated in the left panel of Figure 2a, where the upper and lower boundaries are bounded by air channels (with trivial topology) while the left and right are imposed as periodic boundaries. There were four edges constructed in this geometry, denoted as E_1_, E_2_, E_3_, and E_4_. The projected band structure of this strip is shown in the right panel of Figure 2a, which indeed presents four edge states emerging in the bulk band gap corresponding to the four edges. The edge states are indicated by the colored curves while the bulk states by the grey shading. Among these four edge states, there were two types. One is the valley edge states, which are localized at the E_2_ and E_3_ edges (see Figure 2b). Due to the biased magnetic fields, the dispersions of the edge states become titled, consistent with the Dirac point tilting shown in Figure 1c. This indicates that the valley edge states are not only valley-dependent, but also carry an anti-chiral signature. The other type of edge state is the edge states localized at the interfaces between PC1 and the air channel (i.e., E_1_ and E_4_, see Figure 2b). Unlike the former, where physical interfaces are constructed to prevent the inter-valley scattering and therefore the edge states are valley-dependent, the latter edge states imprint only the anti-chiral signature while lacking valley-dependence. 

It has been shown that in valley crystals, the valley edge gaps often permit nontrivial higher-order topology that supports topological corner states [33,34]. To see the corner states in our valley crystal, we constructed a triangular-shaped box geometry consisting of PC2 surrounded by PC1 (formed by E_3_-type edges), as schematically illustrated in the inset of Figure 2c. The reason that we chose the E_3_-type is that it has a relatively large edge gap (see Figure 2a, the light-red shading) that can stabilize the corner states. The eigen-spectra of the box geometry is shown in Figure 2c, with grey, red, and purple symbols indicating the bulk states, the E_3_ valley edge states, and the in-gap corner states, respectively. Figure 2d presents the electric field distributions of the three in-gap states, which show typical behaviors of corner states (i.e., the energy is tightly confined at three geometric corners and decays exponentially into both the edge and bulk regions). It is worth mentioning that although the corner states emerge as the manifestation of the nontrivial valley Hall effect (which is originated from and protected by the $C_{3v}$ crystalline symmetry), they are still affected by the biased magnetic fields. As a consequence, the corner states are no longer degenerate and become slightly titled (see Figure 2c). This was similar to the valley edge states E_2_ and E_3_, only that when compared to the propagating edge states, the localized corner states were less affected by the magnetic bias. This can be seen from the high localization of the corner states in Figure 2d. 

## 4. Reconfigurable Light Imaging

The uniqueness about our system is that the corner states are bounded to fall in the frequency regimes of the E_2_ and E_4_ anti-chiral edge states (whose frequencies are also largely overlapped, as shown in Figure 2a). This means that we can skillfully exploit different combinations of edge and corner states to realize versatile reconfigurable functionalities. It is known that waveguides and resonators are two types of basic elements to realize large-scale integrated devices, which have a wide range of applications in lasing, signal filtering, and sensing [48,49,50]. Here, we designed a waveguide-resonator structure, as shown in Figure 3a, where a triangular whisper-gallery ring formed by the E_3_ edges was evanescently coupled to an E_4_-edge port. The edge port acts as a 1D waveguide channel that imprints the anti-chiral signature of the E_4_ edge states. First, we considered an incoming source from the left port-entrance with a frequency of 5.87 GHz, which cut through both the E_2_ and E_4_ edge dispersions but fell outside the corner state frequencies. It is shown in Figure 3b that the rightward propagating waves are excited in the E_4_-port, which, upon reaching the ring resonator, light up the triangular whisper-gallery mode. Despite the evanescent coupling, substantial energy from the waveguide was coupled to the resonator, suggesting a high efficiency.

When we increased the operating frequency of the waveguide-resonator device to coincide with the corner states at 5.93 GHz, surprisingly, it was observed that despite the fact that this frequency also cut through the E_2_ edge dispersion, the incoming wave from the edge port only excited the corner states in the ring resonator (see Figure 3c). This is due to the large quality factor known for the topological corner states [51,52]. More interestingly, Figure 3c shows that the anti-chiral E_4_ edge states can go around the lower-right sharp corner to illuminate the upper corner of the triangular ring, indicating their topological robustness. This is seldomly observed in traditional waveguides that reflect most of the incoming energy upon sharp turns. By continuously tuning the operating frequency, other whisper-gallery modes can be excited, demonstrating an interesting broadband multiplexing functionality in one single device, which is otherwise difficult to realize in conventional waveguide-resonator systems. 

Before demonstrating more complex designs, we first studied the robustness of the topological states in our system, which included three types. One consists of the anti-chiral edge states induced by breaking the time-reversal symmetry (i.e., the E_1_ and E_4_ edge states). The other two types including the valley-dependent edge states (i.e., the E_2_ and E_3_ edge states) and the corner states come from breaking the inversion symmetry. 

For the anti-chiral edge states due to the breaking of time-reversal symmetry, they were rather robust to various non-magnetic perturbations [16,17]. To see this, we deliberately introduced three types of perturbations including expanding/shrinking some random cells by 10% on the edge (see Figure 3d), inserting a rectangular non-magnetic obstruction with a dielectric constant of 12 and the geometric size of 0.24a×0.6a (see Figure 3e), and removing some PC rods (see Figure 3f). Excitation studies (similar to Figure 3c) have been conducted to see how these perturbations affect the propagation of the anti-chiral edge states. As expected, regardless of the types of perturbations, the anti-chiral edge states are seen to be rather robust and can always go around the perturbations, maintaining their propagation. Accordingly, the excitation of the corner states in the triangular ring resonator is hardly affected, as shown by the brightness of the three corners in Figure 3d–f. 

Next, we studied the robustness of the valley edge and corner states. As above-mentioned, these types of topological states are protected by the $C_{3v}$ crystalline symmetry. As a consequence, they are only robust against $C_{3v}$-preserving perturbations but can be distorted if the perturbations break the $C_{3v}$ symmetry. This is a typical property for the topological states induced by crystalline symmetries. We also conducted simulations to verify this property. As shown in Figure 3g–i, the same types of perturbations as that in Figure 3d–f were introduced on the interface between PC1 and PC2 (where the valley-dependent edge states are localized). It was seen that expanding/shrinking unit cells and inserting an obstruction barely affected the propagation of the valley edge states (Figure 3g–h). This is because the former respects the $C_{3v}$ symmetry and in the latter, the obstruction was small and had a negligible effect on the background lattice symmetry (however, if the obstruction becomes larger, it will distort the valley edge states). On the other hand, removing some PC rods breaks the $C_{3v}$ symmetry and indeed, the valley edge states are distorted, as shown in Figure 3i. 

For the corner states, a similar robustness property as the valley edge states was observed. As shown in Figure 3j–l, $C_{3v}$-preserving perturbations had a negligible effect on the excitation of the corner states while $C_{3v}$-breaking perturbations severely distorted the corner states and hindered their excitation. It was pointed out that considering the strong localization of the corner states, the perturbation strength for the corner states was chosen to be smaller than that for the edge states. Specifically, the shrinking of the unit cells was taken as 5% and the obstruction was set as 0.18a×0.4a.

The above studies show that our edge and corner states are robust as long as the perturbations are local and (largely) preserve the $C_{3v}$ symmetry. Such a robustness, together with other interesting properties including the large quality factor of the corner states and their controllable excitation based on the anti-chiral edge states, further inspires us to design more complex light imaging that can be tuned and reconfigured. As shown in Figure 3m, we designed a complex pattern depicting a pine tree by using PC2 as the digital coding units and PC1 as the background. Similarly, this pattern is attached to an E_4_-port, where the incoming wave is from the left entrance. The numerical simulations show that the incoming signal can illuminate all the PC2 pixels, which image a pine tree (see Figure 3n). As discussed above, the anti-chiral feature of the edge states comes from and is controlled by the magnetic bias. If we flip the magnetic fields, the dispersion tilting will be in the opposite direction (i.e., the dispersion slope will change from positive in Figure 2a to negative). As a consequence, the rightward propagation will not be allowed any further. Indeed, as shown in Figure 3o, upon magnetic field flipping, the incoming wave was fully reflected and the PC2 pixels were turned off. Here, we point out that by changing the input source from the left port-entrance to the right port-entrance (without flipping the magnetic fields), the same tuning effect can also be realized. In addition to the corner state frequency, the reconfigurable imaging can also be demonstrated at other frequencies, which cut through both the E_2_ and E_4_ edge dispersions, taking advantage of the phenomena reported in Figure 3b, only that the excitation may be less efficient due to the general lower quality factor of the edge states compared to the corner states. 

In addition to exploiting the E_4_-type anti-chiral edge states as the control ports, the E_2_-type edge states are also suitable candidates as control ports, given that this type of edge state also carries an anti-chiral signature. To demonstrate this, we designed a more complex two-heart pattern where the PC2 pixels as coding units are arranged in a curved manner to form two heart shapes connected by a waveguide of E_2_-type edge, as illustrated in Figure 4a. The light imaging for this pattern can present even more versatile reconfigurability. If the input signal is at the left port-entrance, both hearts are lit up (see Figure 4b). Upon magnetic field flipping, the PC2 pixels are either completely turned off (see Figure 4c) or could again be fully illuminated from the right port-entrance (see Figure 4d). On the other hand, if we put the excitation source at the center of the edge port, only part of the pattern (i.e., the right heart) could be illuminated, leaving the other half (i.e., the left heart) completely dark, and vice versa with reversed magnetic fields, as shown in Figure 4e,f. 

The above results revealed a surprising yet interesting fact that the corner states and their excitations are no longer limited to the corners of simple geometries such as squares, hexagons, and triangles, instead, they can appear in arbitrary positions as required. Using such a strategy, it is possible to realize reconfigurable light imaging of any shape made of straight lines, corners, curves, etc., with high potentials in optical imaging and switches, among many other fields.

## 5. Conclusions

In conclusion, we proposed and numerically verified versatile reconfigurable light imaging in a photonic HOTI based on the modified Haldane model with broken inversion symmetry. The unique design of our system allows for simultaneous access to both anti-chiral edge states and valley-polarized corner states. Using corner states with high quality factors as coding pixels and anti-chiral edge states that can be controlled by external magnetic fields as control ports, we showed that various tunable and reconfigurable light imaging could be realized. A waveguide-resonator device and two pixel patterns of a pine tree and a two-heart network were proposed where the HOTI showed high potentials of constructing arbitrary complex imaging patterns. Therein, it is controllable whether and where the pixels are turned on or off and correspondingly, the imaging patterns can be selectively illuminated or darkened as required. Our results showed high potentials of higher-order topological states, combined with controllable ports, in modern digital and intelligent ears for applications of future reconfigurable optical imaging, patterned lasing, topological switching, and integrated photonics. In addition, the use of magnetic fields provides feasible and convenient methods for efficient and robust light control and the implementation of the external edge ports enables ready compatibility with various integrated devices.

## Data Availability

The data presented in this study are available on request from the corresponding authors.
